# Plant Bioactive Compounds at the Interface of Extraction Science, Green Nanoparticles and Applied Biotechnology: A Narrative Review

**DOI:** 10.3390/molecules31081351

**Published:** 2026-04-20

**Authors:** Cristina-Ștefania Gălbău, Lorena Dima, Andrea Elena Neculau, Marius Irimie, Lea Pogačnik da Silva, Oana Bianca Oprea, Liviu Gaceu, Mihaela Badea

**Affiliations:** 1 Faculty of Medicine, Transilvania University of Brasov, No. 56, Nicolae Bǎlcescu St., 500019 Braşov, Romania; cristina.adochite@unitbv.ro (C.-Ș.G.); lorena.dima@unitbv.ro (L.D.); andrea.neculau@unitbv.ro (A.E.N.); marius.irimie@unitbv.ro (M.I.); 2 Biotechnical Faculty, University of Ljubljana, Jamnikarjeva 101, 1000 Ljubljana, Slovenia; lea.pogacnik@bf.uni-lj.si; 3 Faculty of Food and Tourism, Transilvania University of Brasov, 500014 Brasov, Romania; oprea.oana.bianca@unitbv.ro (O.B.O.); gaceul@unitbv.ro (L.G.); 4 Research Center for Eco-Biotechnologies and Equipment in Food and Agriculture, Institute for Research and Development, Transilvania University of Brasov, 500484 Brasov, Romania; 5 CSCBAS&CE-MONT Centre/Ince-Romanian Academy, Casa Academiei Române, Calea 13 Septembrie No. 13, 050711 Bucharest, Romania; 6 Academy of Romanian Scientists, Ilfov Street, No. 3, 050044 Bucharest, Romania

**Keywords:** green synthesis, plant-based nanoparticles, nanobiotechnology, in vitro models, biomedical applications

## Abstract

In the contemporary era, nanotechnology has become a central pillar in numerous domains, particularly in cosmetics, nanoelectronics, nanomedicine, and nanobiotechnology. Defined by its focus on materials with dimensions ranging from 0.1 to 100 nm, nanotechnology offers unique physicochemical properties—such as enhanced reactivity, conductivity, and permeability—attributable to the nanoscale. These properties facilitate greater interaction with biological systems, notably improving cellular uptake and functional efficacy. The increasing demand for eco-friendly and biocompatible nanomaterials has driven interest in green synthesis routes, particularly those utilising plant extracts. These methods stand out due to their low toxicity and environmental impact, positioning it as a safer alternative to conventional chemical or microbial methods. Plant-extract-mediated nanoparticles demonstrate promising applications in diagnostics, drug delivery, regenerative medicine, and neurotherapeutics. Their role in precision medicine, including gene and drug delivery and the imaging of neurological disorders, underscores green nanotechnology’s transformative potential. This review highlights recent advances in the synthesis, functionality, and biomedical applications of plant-based nanoparticles, emphasizing their relevance in in vitro models and prospective clinical settings.

## 1. Introduction

We are currently living in an age where most aspects of everyday life have a nano-focus [1]. The main focuses of nanotechnology are the cosmetics industry, nanoelectronics, nanomedicine and nanobiotechnology [2,3,4].

This new technology involves the study and use of materials with very tiny structures and scales ranging from 0.1 to 100 nm [5]. Due to their small size, these nanomaterials exhibit properties distinct from those of bulk materials, including chemical reactivity, electrical conductivity, physical resistance, magnetism, and optical effects [6,7,8,9]. Due to their nanometric dimensions, the passage of these particles through the biological membrane and their influence on cell physiology are more accessible, as a decrease in diameter increases the contact surface, directly influencing particle penetration and efficacy [10,11].

The search for new materials and methods to synthesise and manufacture them is increasing, making nanotechnology the fastest-growing area of manufacturing [12]. Nanoparticles (NPs) naturally occur within both organic and inorganic compounds, originating from processes such as microbial activity, forest fires, and plant-mediated phytoremediation. Applying this new technology, significant progress has been made in developing new methods for the preparation and use of nanomaterials. In laboratory settings, nanoparticles can be synthesised using physical, chemical, or biological approaches, employing a wide range of sources, including chemical reagents, polymers, proteins, plant extracts, and microorganisms [13,14,15].

Several authors report the use of bacteria, fungi, yeasts, and plant sources for the biological synthesis of NPs. Green synthesis, as the synthesis of NPs derived from plant extracts is commonly known, has several advantages over microbial synthesis, as it significantly reduces the risk of endangering humans and the environment [16,17]. Numerous in vitro studies attest to the residues’ status as dependable suppliers of useful compounds with a wide range of biochemical functions. Significantly, the experiments also show that green extraction techniques can reduce environmental impact while yielding yields on par with traditional extractions [18]. Together, the prebiotic, enzyme-inhibitory, anti-inflammatory, and antioxidant properties found in various residue sources support their potential for use in formulations targeting the microbiota, functional foods, and nutraceuticals [19].

Extract-based nanoparticles have well-established applications in medicine, including in vivo imaging, in vitro diagnostics, tissue engineering, regenerative medicine, and biomolecular detection [20]. The most significant applications include the detection and treatment of various illnesses via biosensors, bioimaging, targeted drug delivery, hyperthermia, and targeted gene and vaccine delivery [21]. Nanoparticles have also been used in the imaging and treatment of brain illnesses, including brain cancer and abnormalities of the central nervous system, which are common but poorly managed diseases [22,23]. The diversity in phytochemical content, which varies depending on species, growth circumstances, and extraction techniques, is a significant drawback of plant-mediated nanoparticle synthesis. Reproducibility across investigations is hampered by this heterogeneity, which leads to notable variations in nanoparticle size, shape, and biological activity. Furthermore, the transfer of these systems from laboratory-scale research to clinical applications is hampered by the absence of standardized methodologies.

This study attempts to objectively evaluate information on plant-mediated nanoparticle generation and their synergistic effects on cultured cells, with an emphasis on molecular pathways, variability in biological response, and existing experimental design limitations.

## 2. Literature Overview and Biological Implications

This section synthesises current evidence from the literature regarding the biological effects of plant-derived compounds and nanoparticles, with an emphasis on mechanistic insights and comparative analysis.

### 2.1. Plant Effects on Culture Cells

The efficacy and biological effects of medicinal plants have been investigated in pharmacological studies and traditional medicine [24]. Flavonoids, alkaloids, terpenoids, phenolic acids, and proteins are examples of bioactive secondary metabolites found in medicinal plant extracts that can act as stabilising and reducing agents during the creation of nanoparticles [25].

A real alternative to mammalian scaffold systems is in vitro plant cultures, which can produce aggregates and generate diverse matrices composed of cellulose, pectin, and hemicellulose, and which lack the human diseases associated with mammalian scaffold systems. Plant cells in culture medium provide high biomass yields, short doubling times, high behavioural uniformity, and low costs. Additionally, plant cell cultures can undergo genetic transformation, enabling them to synthesise a wide range of functional proteins for use in human pharmaceuticals, including growth factors, vaccines, monoclonal antibodies, and cytokinins [26,27].

Plants adapt to both biotic and abiotic stress through the production of secondary metabolites [28]. These compounds are typically induced by external stressors, such as oxidative stress, mechanical injury, or pathogen attack, and are mediated by signalling molecules, including jasmonates, salicylic acid, and their derivatives [29]. Biotic and abiotic elicitors modulate gene expression in response to chemical or physiological stimuli, influencing enzyme activity and facilitating the biosynthesis of secondary metabolites, including flavonoids, alkaloids, terpenoids, thionins, phenylpropanoids, and polypeptides [30]. While these metabolites serve protective roles for the plant, many also exhibit significant medicinal properties in humans [31].

A sustainable and environmentally friendly process, plant-mediated green synthesis of metallic and metal oxide nanoparticles uses bioactive plant constituents such as flavonoids, alkaloids, terpenoids, phenolic acids, sugars, and proteins as natural reducing, capping, and stabilising agents during the nanoparticle formation process, in place of hazardous chemical reducers and stabilisers [32]. By facilitating nucleation, donating electrons to metal ions, and regulating growth kinetics, these phytoconstituents create nanoparticles with controllable size and shape and improved surface functioning [32]. Using alcoholic or aqueous plant extracts from leaves, stems, fruits, and peels at mild pH and temperature, green synthesis techniques have been effectively applied to a variety of metal nanoparticles (Ag, Au) and metal oxide systems (ZnO, FeO_4_) [33].

Recent experiments have shown that zinc oxide (ZnO-NPs) and silver (AgNPs) nanoparticles prepared from plant extracts exhibit significant biological activities in vitro. These activities include dose-dependent decreases in the viability of cancer cells as determined by common tests (for example, MTT, MTS), as well as modifications of oxidative stress responses, antimicrobial effects, and selective cytotoxicity against tumor cell lines while preserving healthy cells [34]. Increased cellular uptake and therapeutic potential can result from adding bioactive secondary metabolites to nanoparticle surfaces, which can also enhance antioxidant properties and modulate cell signalling pathways. These findings support the development of green-synthesised nanoparticles as attractive candidates for biomedical and therapeutic applications by indicating that plant-derived phytochemicals not only mediate nanoparticle formation but also modify subsequent cellular responses [35]. Despite their therapeutic potential, there is limited research on industrial-scale technologies for the production and extraction of pharmaceuticals from plant cell cultures [36]. Notable examples include: shikonin, an anti-inflammatory and anti-tumor compound from *Lithospermum erythrorhizon* [37]; berberine, an antimicrobial alkaloid found in *Coptis japonica* and *Coscinium fenestratum* [38]; rosmarinic acid, a potent antioxidant and anti-inflammatory agent from *Coleus blumei* [39]; sanguinarine, an anti-inflammatory alkaloid produced by *Eschscholzia californica* [40]; and vogonosin, a flavone from *Scutellaria baicalensis*, noted for its strong apoptotic activity that selectively targets cancer cells while sparing healthy ones [41]. In addition to being bioactive compounds with proven anticancer properties, compounds like berberine and shikonin may also serve as functional elements in plant extracts used to create nanoparticles. During green synthesis, these phytochemicals may act as stabilizing or reducing agents, affecting the characteristics of nanoparticles and the ensuing interactions with cells. Their inclusion in this review emphasizes how plant-derived chemicals play a dual role as important contributors to the production and functionality of nanoparticles as well as therapeutic medicines.

The majority of incurable illnesses and cancers are linked to elevated levels of oxidative stress [42]. Oxidative stress can harm several physiological and biochemical functions. Overproducing these free radicals may also harm macromolecules, including proteins, lipids, and DNA, via oxidative damage [43]. Numerous chronic illnesses, such as cancer, atherosclerosis, diabetes, aging, and other degenerative disorders in people, may ultimately result from this process [44].

Due to phenolic components (for example, quercetin [45], kaempferol [46], epigallocatechin gallate [47], etc.), most medicinal plants with anticancer properties also have antioxidant activity. Thus, these plants may combat free radicals to achieve their anticancer effects, either wholly or partially. Hydroxyl groups in phenolic substances, such as flavonols and flavonoids, contribute to their antioxidant properties [48,49]. Scavenging free radicals is a critical function of therapeutic herbs with antioxidant activity. Additionally, they can lessen the toxicity of harmful substances that cause oxidative stress [43,50]. If this is the case, further research should be conducted to determine whether additional plants with antioxidant properties also exhibit anticancer properties. However, a key indicator of cytotoxic anticancer drugs is their ability to induce apoptosis. Research studies have shown that apoptosis is the mechanism by which alkaloids and phenols exert their deadly impact on many tumor types. Phenolic chemicals have antitumoral effects via influencing processes related to cell proliferation (such as G2/M cell cycle arrest and suppression of topoisomerase II), angiogenesis, apoptosis, and the pathways of protein kinase B (Akt) and phosphoinositide 3-kinase (PI3-K). Research has shown that suppressing PI3-K activity inhibits Akt and mTOR phosphorylation, ultimately decreasing nuclear factor-κB (NF-κB) activity. This process reduces the development of cancer cells and enhances cell death by inhibiting transcription and creating the proteins that drive the cell cycle [51,52]. For instance, the phenolic quercetin found in plants inhibits Akt and IKKα- phosphorylation, which stops the transcription factor NF-κB from being activated and has the consequent anticancer effect [53]. By altering several cellular pathways linked to cancer, phenolic phytochemicals such as flavonoids and polyphenolic acids have shown notable antitumoral benefits. For example, by regulating the miR-224-3p/PTEN axis, the flavonol quercetin has been shown to suppress the PI3K/Akt signalling pathway, resulting in reduced proliferation and enhanced apoptosis in children’s acute myeloid leukaemia cells [54]. Likewise, in breast cancer cell lines, quercetin inhibits Akt/mTOR/PTEN signalling and induces apoptosis, which lowers cell growth and survival [55]. The phenolic acid p-coumaric acid inhibits the activity of the PI3K/Akt pathway and causes apoptosis and G2 phase cell cycle arrest in osteosarcoma models, which reduces the rate of increase of tumor cells [56]. Furthermore, curcumin and its derivatives target PI3K/Akt signalling and enhance pro-apoptotic mechanisms to produce antitumor effects; novel curcumin derivatives have been shown to interfere with the PI3K/Akt/p53 axis, leading to G2/M arrest and apoptosis in MCF-7 breast cancer cells [57]. Taken together, these studies demonstrate that phenolic compounds can affect key mechanisms linked to cell division, death, and survival signalling, such as the PI3K/Akt pathway, supporting their anticancer potential.

### 2.2. Nanoparticles’ Effects on Culture Cells

Cell culture allows the dissection of complex biological events into smaller components, albeit in an artificial environment separate from the multifactorial environment of the body’s organs and tissues. Nanoscale materials differ in their properties from those of materials at the microscale or larger. NPs have a large specific surface area, high surface reactivity, strong particle interactions, and electrical and optical properties, as well as superb electrocatalytic and photocatalytic properties, making them versatile for applications in energy, the environment, food, medicine, etc. In biomedicine, NPs are used as carriers for drugs, vaccines, and other therapeutics. For this reason, research has been conducted to identify multiple nanoparticle configurations that can readily interface with living cells, owing to their size compatibility and unique, exploitable therapeutic properties [58,59].

Eom & Choi [60] reported that CeO_2_ nanoparticles induced oxidative stress in cultured cells. Plant extracts contain polyphenolic chemicals that can alter redox-sensitive pathways, especially via activating Nrf2 and upregulating downstream antioxidant enzymes such as heme oxygenase-1 (HO-1) [60]. Meanwhile, other studies indicate that CeO_2_ NPs exhibit antioxidant activity and reduce oxidative stress [61]. Thus, the biological effects characteristic of nanoparticles’ physical and chemical properties are made explicit. The Ce^3+^/Ce^2+^ ratio, particle size, and the surrounding microenvironment are some of the physicochemical characteristics that may be responsible for the dual antioxidant/pro-oxidant behavior seen for CeO_2_ nanoparticles. While differences in surface chemistry and biological context may tip the scales in favor of pro-oxidant activity, smaller particles with bigger. Ce^3+^ percentage is often linked to increased radical scavenging capacity [62].

According to Mühlfeld [63], NPs primarily interact with biological environments at the cellular level through structural and functional cellular compartments, such as cell membranes, the nucleus, and organelles. The chemical makeup of the nanomaterial is the primary factor determining how it interacts with the cell. Furthermore, four key aspects of nanoparticles affect cells: first, their absorption by cells; second, their release of metal ions; third, their surface reactivity; and fourth, their adsorption capacity. Numerous particle properties, including size, shape, texture, stiffness, charge, the presence of functional groups, and hydrophobicity/hydrophilicity, among others, affect cellular uptake and interactions with cellular components [64,65].

Clathrin-mediated endocytosis is one of the most critical pathways in the uptake of NPs (for example Au, SiO_2_) [66] by cells (for example HeLa cells, macrophages and epithelial cells) [67], being able to take up submicron particles with a size of 100–350 nm [68]; in turn, there are reports that nanoparticle uptake can occur through caveolae-mediated endocytosis, which is responsible for cellular uptake of NPs (20–100 nm). Endocytosis of nanoparticles depends not only on the particle but also on the cell type; for example, compared with phagocytic cells (macrophages), epithelial cells use different uptake mechanisms [69,70].

Reports of nanometre-sized particles in cells, both in vivo and after cell culture, are available. It has been suggested that nanoparticles may be internalised by pinocytosis. Pinocytosis refers to the ingestion of fluids and solutes by vesicles of about 100 nm in diameter [71,72]. However, Stearns et al. [73] reported that upon culturing a human epithelial cell line with TiO_2_ NPS, the nanoparticles, which separated from the cells, formed a layer on the cell surface. On the other hand, they reported the presence of ultrafine particles with diameters less than 100 nm and 0.21 μm polystyrene microparticles after cell culture in macrophages; the uptake of the microparticles was not blocked by cytochalasin D, whereas that of the ultrafine particles was. Since the nanoparticles were not bound to the membranes, their uptake is assumed to be non-phagocytic [74].

The harmful effects of nanoparticles are often caused by their internalisation into cells [75]. However, for many biomedical applications of nanomaterials to succeed, the materials must enter the cell. Consequently, it’s critical to understand nanoparticles’ cellular uptake mechanisms before delving further into their cytotoxicity, cellular metabolism, and cytotoxicity mechanisms. Additionally, this will help create therapeutic nanomaterials that are safer for the environment and have improved cellular targeting and absorption capabilities [76,77].

Nanoparticles, when submerged in a biological fluid, are exposed to a medium different from that used during manufacture. The nanoparticles will be forced to interact with the surrounding media, potentially altering their chemical and physical properties [78].

Instead of maintaining their synthetic surface identities, nanoparticles introduced into biological systems selectively adsorb surrounding proteins, lipids, and other endogenous biomolecules, forming a dynamic biomolecular corona that controls physiological distribution, cellular uptake, and subsequent biological recognition [79]. The phytochemical profile of the plant extract in green-synthesised systems determines the surface chemistry of the nanoparticles, which has a significant impact on the composition of the protein corona. While protein-rich extracts may result in a distinct corona composition and modified biological responses, polyphenol-rich coatings may promote selective adsorption of serum proteins, thereby improving cellular uptake. Proteins, including serum albumin, apolipoproteins, and acute phase proteins, interact with a variety of lipid species in this layered corona, including phospholipids and ceramides [80,81]. Recent research indicates that nucleic acids and tiny metabolites may also be incorporated into the corona structure. The physicochemical characteristics of the nanoparticle and the biofluid environment determine the structure and composition of this corona, which, in turn, alters the nanoparticle’s effective identity and therapeutic efficacy [82,83].

NPs may be absorbed by cells via energy-free mechanisms, such as translocation or simple diffusion. The majority of NP absorption routes, however, rely on endocytosis for energy. The process of endocytosis involves the formation of vesicles from the plasma membrane that transport materials from the extracellular to the intracellular environment, including particles, nutrients, and dead cells [84]. Phagocytosis and pinocytosis are the two categories under which endocytosis is classified.

Phagocytosis is the cellular ingestion of particles (0.5–10 μm) in the plasma-membrane envelope. It is a host defence mechanism since it absorbs and internalises foreign substances, including dust, dead cells, and cell debris [77,85]. This method is ligand-induced; NPs are taken up by opsonins that bind them and engage cell-surface complement receptors [86].

The process by which cells absorb dissolved solutes and extracellular fluids is known as pinocytosis [87]. Endocytosis can be classified into three main pathways: receptor-mediated endocytosis, macropinocytosis, and clathrin- and caveolae-independent endocytosis. The latter is further subdivided into clathrin-dependent and caveolae-dependent mechanisms based on the involvement of specific proteins [88].

### 2.3. Synergetic Effects of Plants and Nanoparticles on Culture Cells

These synergistic effects originate from multi-target synergistic effects, modulation of pharmacokinetic or physico-chemical effects, interference with resistance mechanisms or elimination and neutralisation potentials [89,90,91,92].

Scientific investigations proved the ability of secondary compounds or plant extracts (artichoke polyphenols (artichoke), chlorogenic acid (coffee), curcumin (curcuma), daidzein (soy), epigallocatechin-3-gallate (green tea), genistein (soy), ginsenoside Rg-3 (ginseng), lycopene (tomato), phenethyl isothiocyanate (brocoli, cabbage, etc.), pterostilbene (blueberries), resveratrol (red grapes, cranberries etc.), sulforaphane (brocoli, cabbage and kale), quercetin (onion, buckwheat and citrus)) to eliminate or neutralize the toxic or secondary effects of a drug [93,94].

There is growing interest in using plant extracts as reducing agents in nanoparticle synthesis. This increasing popularity is attributed to their biocompatibility, scalability, and the availability of straightforward synthesis protocols, which collectively make plant-based approaches more appealing [95]. In cell culture, the main focus is on producing commercially important bioactive compounds, which is why their production is enhanced by using tissue culture technology [96].

Plants comprise various active phytocompounds, which can be divided into primary and secondary metabolites. The primary metabolites include vitamins, proteins, amino acids, nucleic acids, polysaccharides, and reducing sugars. Secondary metabolites are flavonoids, volatile oils, carotenoids, terpenoids, coumarins and alkaloids [97]. Current studies are mainly focused on obtaining secondary metabolites from cell/tissue culture of medicinal plants; the aim of utilising NPs to obtain these bioactive compounds is to achieve a considerable increase in the therapeutic uses of these plants [98,99]. Secondary metabolites exhibit diverse biological properties, including anti-inflammatory, antibacterial, antifungal, anticancer, and antioxidant activities (Table 1). There were reports of enhanced immune function/immunomodulation, UV protection of the skin, rejuvenating properties, collagen production, and the treatment of psoriasis [100,101,102].

At the same time, biomolecules closely linked to the synthesis confer functionality to nanoparticle surfaces, providing synergistic effects for antimicrobial applications or cancer treatments and reducing toxicity to higher organisms [103]. Several investigations have been conducted to improve the production of bioactive compounds, with a focus on titanium dioxide (TiO_2_) NPs. There are comparative reports on the effects of NPs on the in vitro production of α-tocopherol from argan using NPsTiO_2_ and NPsSiO_2_ as elicitors, where both particles outperformed the control by 4.59 and 4.7 times, respectively [104]. Kruszka et al. [105] reported how Ag NPs can change metabolites in *Arabidopsis thaliana* due to differences between variants, effects over time, and interactions between variants and time. The above research reports illustrate the synergistic effects on secondary metabolite biosynthesis, which will depend on the nanomaterial used, the plant species and the doses [106].

A summary of a few works in the field is given in Table 1; however, due to considerable differences in experimental circumstances, nanoparticle manufacturing procedures, and biological models, direct comparisons between systems are difficult. Because of this, quantifiable metrics like zeta potential or IC_50_ values are reported inconsistently between research and should be evaluated in the context of each unique experimental setting.

It should be mentioned that silver nanoparticles are widely studied in green synthesis techniques, which explains why they are so prevalent in the literature. However, research on other nanomaterials such as TiO_2_ and gold nanoparticles shows that this area of study is becoming more broad and varied.

**Table 1:** Nanoparticles synthesised by green methods and their effects on cell cultures.

Nr Crt	Plant Type	Extraction Method	Nps Type	Synthesis Method of NPs	Types of Tests for NPs	Particle Size (nm)	Zeta Potential (mV)	Type of Cells Culture	Cell Culture Tests	IC_50_	Ref.
1	*Achillea Millefolium*	3 g plant in 100 mL water, 100 °C, 20 min	Ag	AgNO_3_ + extract, ultrasonication (15 min), 40 °C, 2 h stirring	FTIR, XRD, SEM;	22.4 ± 7.4	-	MOLT-4 (human lymphoblast; acute lymphoblastic leukaemia)	MTT assay	0.011 μg/mL	[17]
2	*Lavandula angustifolia*	1 g/100 mL water, microwave 1 min	Ag	Extract + 5 mM AgNO_3_, microwave 1 min	-	<100	-	U87MG human brain tumor cells	MTT assay, caspase activation assay, Western blot analysis	7.536 μg/mL	[107]
3	*Cannabis sativa*	10 g/100 mL water, boiled 30 min	Au Ag	Extract + HAuCl_4_ or AgNO_3_ (1–10 mM), 60–100 °C, 1–10 min	TEM, SAED, AFM, zeta potential, FTIR, ICP-MS;	12–18	−12.3	*Escherichia coli*, *Pseudomonas aeruginosa* and *Staphylococcus epidermidis*	MIC, MBC	-	[108]
4	*Mentha piperita*	25 g/100 mL water, boiled 30 min	Ag	Extract added to 1 mM AgNO_3_ (V = 100 mL, pH 9).	FESEM. EDS, XRD and thermogravimetric;	15–50	-	*Escherichia coli* and *Staphylococcus aureus*	MIC, MBC	-	[109]
5	*Aloe barbadensis miller*	100 g/500 mL water, boiled 90 min	Ag	Extract added to 1 mM AgNO_3_ (pH 9)	FESEM. EDS, XRD and thermogravimetric;	10–22	-	*Escherichia coli* and *Staphylococcus aureus*	MIC, MBC	-	[109]
6	*Cymbopogon citratus*	50 g/200 mL water, boiled 30 min	Ag	Extract added to 1 mM AgNO_3_ (pH 9)	FESEM. EDS, XRD and thermogravimetric;	10–50	-	*Escherichia coli* and *Staphylococcus aureus*	MIC, MBC	-	[109]
7	*Coriandrum sativum*	23 g/100 mL water, boiled 30 min	Ag	Extract added to 1 mM AgNO_3_ (pH 9)	FESEM. EDS, XRD and thermogravimetric;	5–37	-	*Escherichia coli* and *Staphylococcus aureus*	MIC, MBC	-	[109]
8	*Chelidonium majus* L.	30% ethanol, cold repercolation, 3 days	Ag-MnO_2_	MnO_2_ + 5 mM AgNO_3_ in extract, 6 h stirring	SEM, TEM, EDX, XRD, FTIR;	32.47 ± 0.73	-	Normal keratinocytes (HaCaT) and skin melanoma (A375)	MTT assay, LDH assay, NO assay	-	[110]
9	*Vinca minor* L.	30% ethanol, cold repercolation, 3 days	Ag-MnO_2_	MnO_2_ + 5 mM AgNO_3_ in extract, 6 h stirring	SEM, TEM, EDX, XRD, FTIR;	10.09 ± 0.14	-	Normal keratinocytes (HaCaT) and skin melanoma (A375)	MTT assay, LDH assay, NO assay	-	[110]
10	*Populi gemmae*	70% ethanol, 24 °C 10 min + ultrasound 50 °C 30 min	Ag	Extract (10 mg/mL) + AgNO_3_ (1–5 M), 2 h stirring	thermogravimetric analysis, FTIR, SEM, STEM;	3–60	-	Lung adenocarcinoma cell line (A549) and human breast adenocarcinoma (MCF7)	MTT assay	-	[111]
11	*Populi gemmae*	70% ethanol extraction + ultrasound 50 °C 30 min	Ag	Extract (10 mg/mL) + AgNO_3_ (1–5 M), 2 h stirring.	thermogravimetric analysis, FTIR, SEM, STEM;	5–150	-	*Streptococcus pyogenes*, *Staphylococcus aureus*, *Escherichia coli, Pseudomonas aeruginosa*, *Candida albicans* and *Candida parapsilosis*	MIC and MBC	-	[111]
12	*Viburnum opulus*	40 g fruits in acetone:water (1:4), 1 h at room temp	Ag	1% AgNO_3_ + extract, boiling 10 min	TEM, XRD, FTIR;	10–50	-	HaCaT, in vivo studies on Wistar rats	ELISA IL-1α and IL-6	-	[112]
13	*Terena asiatica*	4.8 g fruit powder in deionized water, stirred 2 h at 40 °C	TiO_2_	Extract + 1.5 N titanium tetraisopropoxide stirred 3 h at room temp	XRD, FTIR, SEM, EDX, HR-TEM, DLS, zeta potential, UV-VIS;	56.65	−11.1	MCF-7 cells	MTT assay, A0/EB fluorescence, Annexin V/PI	120 μg/mL	[113]
14	*Tridax procumbens*	Aqueous plant extract	TiO_2_	*T. procumbens* extract-mediated green synthesis	UV-VIS, FTIR, XRD, SEM;	24.36	-	KB cells	MTT assay; DAPI staining; apoptotic gene expression	61 µg/mL (KB; 24 h)	[114]
15	*Thalassia hemprichi*	Seagrass extract by hot plate combustion method (HPCM)	TiO_2_	Seagrass extract-mediated green synthesis via HPCM	UV-VIS, FTIR, XRD, SEM, EDX;	50–78	-	MCF-7 cells	MTT assay	64.14 µg/mL	[115]
16	*Nasturtium officinale* L.	10 g plant in 100 mL methanol; 70 °C; 1 h;	Au	90 mL 1 mM HAuCl_4_·3H_2_O + 10 mL methanolic extract; room temperature; centrifuged 30 min at 7500 rpm; washed 2× ultrapure water; pellet dried at 60 °C	UV-VIS, SEM, TEM, EDS, FT-IR;	56.4	-	A549 cells	MTT assay Annexin V-FITC/7-AAD flow cytometry; cell cycle PI staining	39.84 µg/mL (A549; 24 h)	[116]
17	*Moringa oleifera*	Methanol extract of seeds	Au	Methanolic seed extract + HAuCl_4_; room temperature;	UV-VIS, TEM	-	-	A549	MTT assay	163.9 ± 13.27 µg/mL (A549)	[117]
18	*Mandragora autumnalis*	Ethanolic leaf extract (MAE)	Au	Ethanolic leaf extract (MAE) + HAuCl_4_; room temperature;	UV-VIS, DLS Zeta potential, SEM, XRD, FTIR;	~500 nm	−19.07	HCT116 MDA-MB-231 Capan-2 22RV1	MTT assay;	MDA-MB-231: 10.0 ± 0.06 µg/mL HCT116: 22.7 ± 0.3 µg/mL Capan-2: 41.1 µg/mL 22RV1: 52.0 µg/mL	[118]
19	*Curcuma longa*	Hydroalcoholic rhizome extract	Au	Hydroalcoholic extract + HAuCl_4_; nanospheres and nanosheets formed; chitosan-coated variant (NCS-TAuNPs) also prepared	UV-VIS, TEM, FTIR, XRD, DLS;	7–29	-	MCF-7 HCT-116	MTT assay	MCF-7: 38.77 ± 3.1 µg/mL HCT-116: 41.26 ± 1.9 µg/mL	[119]
20	*Artemisia absinthium*	10 g powder + 200 mL deionized water; heated 60 °C 10 min; stirred 1 h at RT;	Au	500 µL aqueous extract + 10 mL 1 mM HAuCl_4_·3H_2_O; pH 4; heated 60 °C	UV-VIS, FT-IR, FE-SEM, DLS, Zeta potential;	65.18	−8.0	HeLa HT-29 MCF-7 OVCAR3	MTT assay; flow cytometry (Annexin V-FITC/PI)	HeLa: 21.16 µg/mL HT-29: 71.24 µg/mL MCF-7: 99.72 µg/mL OVCAR3: 45 µg/mL	[120]
21	*Morus nigra*	10 g powder + 200 mL deionized water; heated 60 °C 10 min; stirred 1 h at RT;	Au	500 µL aqueous extract + 10 mL 2 mM HAuCl_4_·3H_2_O; pH 4; heated 60 °C	UV-VIS, FT-IR, FE-SEM, DLS, Zeta potential;	69.13	−26.6	HeLa HT-29 MCF-7 OVCAR3	MTT assay; flow cytometry (Annexin V-FITC/PI)	HeLa: 23.37 µg/mL HT-29: 66.36 µg/mL MCF-7: 191.85 µg/mL OVCAR3: 58.33 µg/mL	[120]
22	*Peganum harmala*	10 g powder + 200 mL deionized water; heated 60 °C 10 min; stirred 1 h at RT;	Au	500 µL aqueous extract + 10 mL 1 mM HAuCl_4_·3H_2_O; pH 4; heated 60 °C	UV-VIS, FT-IR, FE-SEM, DLS, Zeta potential;	217.80	−19.8	HeLa HT-29 MCF-7 OVCAR3	MTT assay; flow cytometry (Annexin V-FITC/PI)	HeLa: 7.69 µg/mL HT-29: 16.66 µg/mL MCF-7: 40 µg/mL OVCAR3: 30 µg/mL	[120]

## 3. Mechanistic Interplay Between Phytochemicals and Cellular Signalling

Over the past two and a half decades, research on plant-mediated nanoparticle synthesis has undergone a remarkable and clearly traceable transformation. In the period 2000–2010, studies were predominantly exploratory in nature, focused on demonstrating the proof-of-concept feasibility of using plant extracts as alternative reducing agents, and on the basic physico-chemical characterisation of resulting nanoparticles—predominantly silver—through techniques such as UV-Vis spectroscopy, XRD, and TEM [13,16]. Between 2010 and 2020, the field expanded substantially in both scope and depth: the diversity of nanoparticle types increased markedly (TiO_2_, ZnO, Au), biological testing became progressively more sophisticated—transitioning from simple MIC assays to MTT cytotoxicity profiling, flow cytometry-based apoptosis assays, and Western blot pathway analysis—and first mechanistic insights into phytochemical-nanoparticle surface interactions began to emerge [20,77,95]. From 2020 to 2026, a clear and accelerating shift toward translational and mechanistic research is apparent, characterised by a growing focus on intracellular signalling pathway modulation (PI3K/Akt, NF-κB, Nrf2/HO-1), biomolecular corona formation, selective cytotoxicity toward cancer versus normal cells, and the urgent need for standardisation of synthesis protocols for clinical applicability. The current research frontier is characterised by multi-functional nanoparticle systems, co-delivery platforms, and the systematic exploration of the bidirectional relationship between plants and nanomaterials—areas that represent the most productive directions for future investigation.

Through the tampering of important cellular pathways, medicinal plant-mediated nanoparticles show strong anticancer, antioxidant, and cytotoxic effects, according to this review of the literature that summarized data from in vitro investigations. Numerous similar findings across various nanoparticle types, plant species, and cancer cell lines are revealed by the evidence base [121].

The most popular method for producing nanoparticles is green synthesis, which uses plant extracts as stabilising and reducing agents. Compared with chemically manufactured equivalents, this environmentally friendly process yields biocompatible nanoparticles with improved therapeutic qualities. Both production and therapeutic action are aided by the bioactive chemicals generated from plants that coat or incorporate into nanoparticles [122].

Broad-spectrum anticancer effectiveness against several cancer types is demonstrated by plant-mediated nanoparticles. Numerous studies have demonstrated selective toxicity toward cancer cells relative to normal cells, with cytotoxic effects that are dose- and time-dependent [123].

A comparative analysis of cytotoxic potency values reported in Table 1 reveals a striking variability across plant-NP systems. Silver nanoparticles derived from *Achillea millefolium* demonstrated exceptionally potent activity against MOLT-4 leukaemia cells (IC_50_ = 0.011 µg/mL) [17], while gold nanoparticles from *Peganum harmala* showed selective cytotoxicity against HeLa cells (IC_50_ = 7.69 µg/mL) [120]—a difference in potency spanning nearly three orders of magnitude. Similarly, TiO_2_ nanoparticles synthesised from *Terena asiatica* exhibited moderate activity against MCF-7 cells (IC_50_ = 120 µg/mL) [113], compared to the considerably more potent response elicited by *Nasturtium officinale*-mediated AuNPs (IC_50_ = 39.84 µg/mL against A549) [116]. These differences cannot be attributed to nanoparticle type alone; rather, they reflect the combined influence of phytochemical composition, nanoparticle size, surface functionalisation, and the intrinsic sensitivity of the target cell line. This underscores a critical gap in the field: the absence of standardised comparative frameworks makes it currently impossible to draw predictive structure-activity relationships across plant-NP systems [32,121].

**Figure 1:**
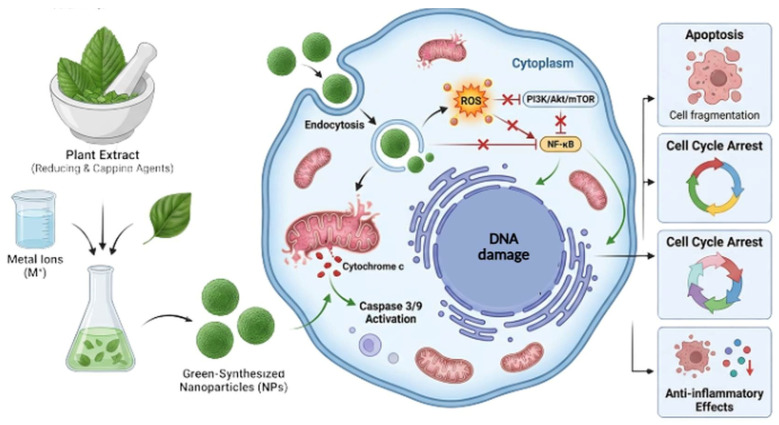
Modulation of PI3K/AKT/NF-κB signalling pathway.

Overall, metal ions and plant-derived capping agents interact intricately to control important signalling pathways like PI3K/Akt and NF-κB, mediating the anticancer effect of green-synthesised nanoparticles. Comprehensive regulation of survival and death pathways is one of the molecular mechanisms driving anticancer actions. Plant-mediated nanoparticles reliably reduce activity in the PI3K/Akt/mTOR pathway (Figure 1), which is often dysregulated in cancer. The tumor environment may be addressed by the anti-inflammatory actions of NF-κB pathway suppression [124].

The main pathways connecting nanoparticle exposure to pathway modification and apoptosis are ROS production and oxidative stress. ROS produced by plant-mediated nanoparticles activate p53, inhibit survival signalling, damage mitochondria, and directly triggers apoptotic machinery. A complex process that can be used for treatment is the capacity to produce therapeutic amounts of ROS in cancer cells while preserving antioxidant qualities in healthy cells or inflammatory conditions [125]. Overall, the anticancer impact of green-synthesised nanoparticles is mediated by complex interactions between metal ions and plant-derived capping agents that regulate key signaling pathways like PI3K/Akt and NF-κB [126].

Beyond additive effects, therapeutic efficacy is increased by synergistic interactions between plant extracts and nanoparticles or between plant-mediated nanoparticles and conventional medications. Enhanced bioavailability and cellular absorption, complementary route targeting, and increased ROS production are all examples of synergism. These results offer compelling evidence in favor of combination therapies for cancer [127].

The physicochemical conditions that nanoparticles experience in biological settings are very different from those that exist during their manufacture and storage. When nanoparticles move from a regulated manufacturing environment into complex biological fluids such as cell culture media, interstitial fluid, or plasma, they must interact dynamically with ions, proteins, lipids, and other biomolecules. Their initial surface chemistry, colloidal stability, hydrodynamic diameter, surface charge, and aggregation state may all be drastically changed as a result. These changes affect circulation time, tissue distribution, and cellular responses, making them more than just physicochemical artefacts. Instead, they are important factors that determine biological performance [128].

**Figure 2:**
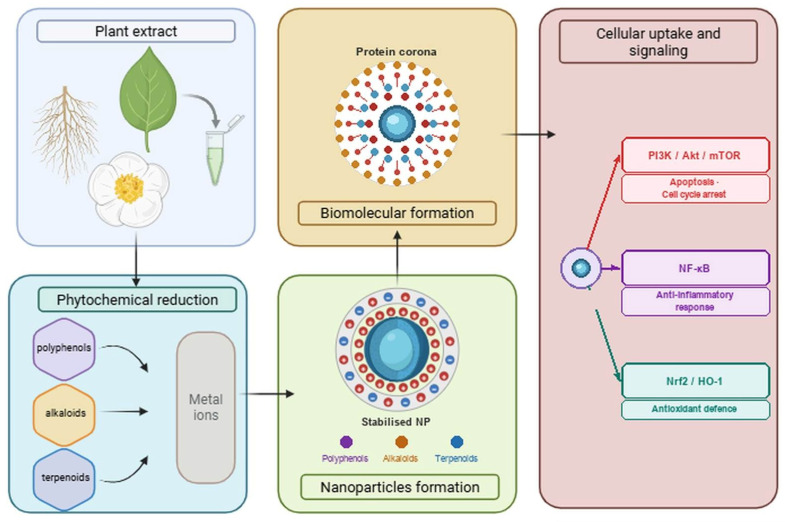
Schematic representation of plant extract-mediated green synthesis of metallic nanoparticles: from phytochemical reduction and capping to biomolecular corona formation and cellular uptake. Key phytochemical classes (flavonoids, alkaloids, terpenoids) donate electrons to metal ions, directing nucleation and surface functionalisation. Upon entry into biological fluids, a dynamic protein/lipid corona forms, reshaping the nanoparticle’s biological identity and governing receptor-mediated internalisation and downstream signalling responses.

The development of the biomolecular corona is a key process that underlies these alterations. Nanoparticles quickly and selectively absorb proteins, lipids, and other endogenous components from the biological environment, creating a dynamic and multilayered corona instead of maintaining their manufactured surface identity. The nanoparticle’s “biological identity,” which may be very different from its synthetic design, is effectively defined by this interfacial structure. Proteins that are commonly recognised as dominant corona constituents include serum albumin, apolipoproteins, immunoglobulins, and acute-phase proteins. At the nano–bio boundary, these proteins form intricate hybrid assemblies with various lipid species, such as phospholipids and ceramides (Figure 2) [129].

Additional layers of compositional and functional complexity may be added to the corona by nucleic acids and low-molecular-weight metabolites, according to new research. Crucially, the composition of the corona is dynamic and depends on both extrinsic environmental parameters (protein concentration, ionic strength, shear stress, and exposure time) and intrinsic nanoparticle attributes (size, surface curvature, charge, hydrophobicity, and surface functionalization) [79,130]. The corona regulates immune activation, receptor recognition, biodistribution, and eventually therapeutic efficacy through this dynamic remodelling. The acquired corona of nanoparticles also significantly affects their cellular uptake. The most common internalisation methods are energy-dependent and require endocytosis; however, limited translocation across membranes may occur via energy-independent processes, including passive diffusion or direct membrane penetration, especially for ultrasmall or extremely lipophilic nanoparticles. The term “endocytosis” refers to a variety of vesicular transport processes in which extracellular material is internalised by the plasma membrane invaginating [131]. Among these, phagocytosis is a specialised technique for the ingestion of large particles (0.5–10 μm) that is mostly carried out by professional phagocytes (such as neutrophils and macrophages) [132]. Opsonisation, in which serum proteins bind to nanoparticles and promote recognition by complement or Fc receptors, frequently aids this ligand-driven process, thereby improving immune clearance [133].

On the other hand, pinocytosis involves multiple mechanistically unique pathways and mediates the uptake of dissolved solutes and nanoscale nanoparticles. Selective internalisation is mediated by receptor-mediated endocytosis through ligand–receptor interactions, which often involve pits coated with clathrin [134]. Certain types of nanoparticles can be more easily absorbed while possibly avoiding lysosomal destruction thanks to caveolae-dependent endocytosis, which is linked to lipid raft domains that are abundant in cholesterol and caveolin proteins. Furthermore, depending on the size, shape, and surface properties of the nanoparticles, internalisation is facilitated by macropinocytosis and clathrin- and caveolae-independent processes [135]. The requirement to incorporate nano–bio interface considerations into logical nanoparticle design is highlighted by the fact that these uptake pathways, when combined with corona formation, define intracellular trafficking routes, endosomal escape potential, and downstream biological effects [136].

The comparative analysis of various plant extracts used in the green synthesis of nanoparticles highlights the significant versatility and potential of botanical resources for the development of biocompatible nanomaterials with antimicrobial [137] and therapeutic [138] activities, as well as for use in cosmetic products [139]. Across the reviewed studies, silver nanoparticles (AgNPs) were the most commonly synthesised, employing aqueous or hydroalcoholic plant extracts through diverse green synthesis protocols, including boiling, sonication, and magnetic stirring. Characterisation techniques such as FTIR, XRD, SEM, TEM, and thermogravimetric analysis confirmed the successful synthesis and stability of the nanoparticles.

Biological assessments revealed promising cytotoxic, antimicrobial, and anti-inflammatory effects of green-synthesised NPs. Notably, plant-NP systems derived from *Achillea millefolium*, *Lavandula angustifolia*, and *Chelidonium majus* exhibited selective cytotoxicity against cancer cell lines (e.g., MOLT-4, U87MG, A375) while maintaining biocompatibility with normal cells (e.g., HaCaT). Furthermore, studies involving microorganisms (e.g., *E. coli* and *S. aureus*) demonstrated significant antibacterial activity, confirming the dual therapeutic potential of plant-based NPs.

Importantly, the synthesis of NPs using plant phytochemicals suggests the possibility of synergistic biological effects. These effects may result from the combined action of metal ions and bioactive plant compounds, enhancing cellular responses beyond the capabilities of each component alone [140]. This synergy underlines the need for future mechanistic studies of the interactions between plant-derived metabolites and metallic nanoparticles at the cellular and molecular levels [141,142].

The standardisation of synthesis methods is one of the most important areas for further study. Reliable manufacturing of nanoparticles with uniform characteristics requires the establishment of repeatable, consistent green synthesis techniques. Standardised protocols will accelerate the optimisation of synthesis processes, facilitate comparison of results across studies, and encourage broader adoption of green synthesis methods in both industry and research [143]. To address safety concerns about green-synthesised nanoparticles, especially for biomedical applications, thorough toxicity studies are essential. The immediate and long-term impacts of these nanoparticles on the environment and human health must be thoroughly examined in future studies. This entails evaluating possible immunogenicity, cytotoxicity, genotoxicity, and biodegradability [143,144].

Finally, the bidirectional relationship between nanoparticles and plant systems remains underexplored—this represents a significant gap and opportunity for future research. Overall, green-synthesised nanoparticles represent a promising, sustainable platform for biomedical applications, though standardisation of extraction and synthesis protocols remains a crucial step toward clinical translation.

## 4. Materials and Methods

### 4.1. Search Strategy and Databases Used

The literature search was conducted across four major scientific databases: PubMed, Scopus, Web of Science, and Google Scholar. Keywords were applied in Boolean combinations (AND/OR) and included: plant cell culture, secondary metabolites, medicinal plants, nanoparticles, cytotoxicity, apoptosis, oxidative stress, and synergistic effects. The search was restricted to studies published between January 2000 and March 2026, in order to capture both foundational research and the most recent advances in plant-mediated nanoparticle synthesis and biomedical application.

### 4.2. Inclusion and Exclusion Criteria

Inclusion criteria were defined as follows: (i) original research articles and review papers published in peer-reviewed journals; (ii) studies reporting in vitro biological activity of plant-derived compounds and/or nanoparticles; (iii) investigations with an emphasis on anticancer, antioxidant, or cytotoxic mechanisms; (iv) articles exploring cellular uptake pathways, modulation of oxidative stress, apoptosis induction, and intracellular signalling (e.g., PI3K/Akt, NF-κB, Nrf2/HO-1); (v) articles published exclusively in English. Studies were excluded if they lacked clear experimental validation, provided insufficient methodological detail, or were not relevant to cellular models. Non-English language articles, conference abstracts, and thesis documents were excluded.

### 4.3. Data Extraction and Thematic Organisation

Data were extracted independently and organised thematically according to three principal directions: (1) the biological effects of plant-derived compounds on cultured cells; (2) the impact of nanoparticles on cellular function and uptake mechanisms; and (3) the synergistic interactions between plant metabolites and nanomaterials. Extracted information included: plant species, extraction method, nanoparticle type and synthesis conditions, characterisation techniques, biological model, assay type, and reported outcomes (e.g., IC_50_, MIC). Findings were analysed comparatively to identify consistent findings, knowledge gaps, and emerging trends relevant to biomedical and biotechnological applications.

## 5. Conclusions

In conclusion, the current research highlights the significant potential of plant-derived compounds, nanoparticles, and green-synthesised nanoparticles in modulating cellular behaviour in vitro. Studies have demonstrated that plant extracts contain a wide array of bioactive compounds with cytotoxic, antioxidant, and antiproliferative properties that can influence cell viability and morphology. Furthermore, the application of nanoparticles in cell culture systems has opened new avenues for targeted drug delivery, cancer therapy, and tissue regeneration. Of particular interest are green-synthesised nanoparticles, which combine the advantages of nanotechnology with the biocompatibility and reduced toxicity associated with plant-mediated synthesis. These eco-friendly approaches not only enhance the therapeutic efficacy but also contribute to the development of more sustainable biomedical applications. Nevertheless, further in-depth studies are required to fully understand the molecular mechanisms underlying their interactions with cellular systems and assess their long-term safety and potential clinical relevance. Although these findings are promising, further mechanistic studies and standardised protocols are necessary to validate these synergistic interactions and ensure their safety and reproducibility in clinical settings.

## Data Availability

No new data were created or analyzed in this study. Data sharing is not applicable to this article.

## Abbreviations

AFM	Atomic Force Microscopy
AgNP	Silver nanoparticles
Akt	Protein kinase B
AuNP	Gold nanoparticles
ELISA IL-1α	Enzyme-Linked Immunosorbent Assay for Interleukin-1 alpha
ELISA IL-6	Enzyme-Linked Immunosorbent Assay for Interleukin-6
FESEM	Field Emission Scanning Electron Microscopy
FTIR	Fourier Transform Infrared Spectroscopy
ICP-MS	Inductively Coupled Plasma Mass Spectrometry
LDH assay	Lactate Dehydrogenase assay
MAPK	Mitogen-Activated Protein Kinase
MBC	Minimum Bactericidal Concentration
MIC	Minimum Inhibitory Concentration
MTS assay	3-(4,5-dimethylthiazol-2-yl)-5-(3-carboxymethoxyphenyl)-2-(4-sulfophenyl)-2H-tetrazolium assay
MTT assay	3-(4,5-dimethylthiazol-2-yl)-2,5-diphenyl tetrazolium bromide assay
NF-κB	Nuclear factor-κB
NO assay	Nitric Oxide assay
Nrf2/HO-1 signaling pathway	Nuclear factor erythroid 2–related factor 2/Heme Oxygenase 1 signaling pathway
NPs	Nanoparticles
PI3-K	Phosphoinositide 3-kinase
P53	Tumor protein p%3
SAED	Selected Area Electron Diffraction
SEM	Scanning Electron Microscopy
TEM	Transmission Electron Microscopy
XRD	X-Ray Diffraction
